# Prevalence and contributing factors to recurrent binge eating and obesity among black adults with food insufficiency: findings from a cross-sectional study from a nationally-representative sample

**DOI:** 10.1186/s40337-021-00509-2

**Published:** 2021-11-25

**Authors:** Rachel W. Goode, Hunna J. Watson, Rainier Masa, Cynthia M. Bulik

**Affiliations:** 1 School of Social Work, University of North Carolina at Chapel Hill, 325 Pittsboro Street, CB #3550, Chapel Hill, NC 27599 USA; 2 Department of Psychiatry, School of Medicine, University of North Carolina at Chapel Hill, Chapel Hill, NC USA; 3 Discipline of Psychology, School of Population Health, Curtin University, Perth, Australia; 4 Division of Paediatrics, School of Medicine, University of Western Australia, Perth, Australia; 5 Department of Nutrition, University of North Carolina at Chapel Hill, Chapel Hill, NC USA; 6 Department of Medical Epidemiology and Biostatistics, Karolinska Institutet, Stockholm, Sweden

**Keywords:** Obesity, Binge eating, Food insufficiency, Black Americans, African Americans, Afro-Caribbeans, Food insecurity

## Abstract

**Background:**

Living in a food-insecure or food insufficient household may increase risk for binge eating and obesity. Because racial disparities in food access, obesity, and access to treatment for disordered eating exist, it is important to examine these relationships in Black populations.

**Methods:**

We conducted a secondary analysis of data from the National Survey of American Life (*N* = 4553), a nationally-representative sample of Black Americans, including African Americans and Afro-Caribbeans. Logistic regression was used to explore the association of food insufficiency with obesity and binge eating.

**Results:**

In the total sample of Black Americans, the prevalence of food insufficiency was 10.9% (95% CI 10.0–11.8%). Food insufficiency was not significantly associated with obesity in Black Americans, but when associations were explored in analyses stratified by ethnicity and sex, food insufficiency significantly predicted an increased odds of obesity in Afro-Caribbeans (odds ratio [OR] = 1.47, 95% CI 1.01, 2.13). Individuals experiencing food insufficiency were more likely to report recurrent binge eating in the last 12 months (3% v 2%, *P* = 0.02) and a lifetime history of binge eating (6% v 3%, *P* = 0.004) compared to those who were food sufficient. After adjusting for socio-demographic factors, food insufficiency was not significantly associated with recurrent binge eating in Black Americans or in sex- and ethnicity-stratified analyses.

**Conclusion:**

The present study reveals a more complex relation between food insufficiency and binge eating than previously thought—although an association existed, it was attenuated by an array of sociodemographic factors. Our results also underscore the importance of considering ethnicity as different patterns emerged between African American and Afro-Caribbean participants.

## Plain English Summary

Living in a food-insufficient household may increase risk for binge eating and obesity. Because we know very little about this relationship in Black Americans, we conducted a research study to examine the rates of food insufficiency, binge eating, and obesity in a sample of African Americans and Afro-Caribbeans. The prevalence of food insufficiency in the entire sample was 10.9%. In Afro-Caribbeans, individuals with food insufficiency were more likely to have obesity, than those who were food sufficient. We did not see this relationship in African Americans. Individuals with food insufficiency were more likely to report recurrent binge eating in the last 12 months, compared to those with food sufficiency. After adjusting for socio-demographic factors, however, food insufficiency was not significantly associated with recurrent binge eating.

## Introduction

Although the majority of U.S. households report having access to enough food to meet their physical needs (e.g., residing in a food secure-home), approximately 37 million Americans live in a food-insecure household [1]. Food insecurity is defined as not having, or being unable to acquire enough food to meet the needs of household members due to insufficient money or other resources for food [1]. A related construct is food insufficiency, a simpler measure of whether or not there was enough food to eat in a household. Considerable overlap exists between the two measures; individuals who suffer from food insufficiency often have the most extreme levels of food insecurity [2, 3]. Factors associated with food insufficiency include lower age, being Black/African American or Latinx race/ethnicity, or being unmarried [3]. Estimates of food insufficiency range from nearly 4–17% in the United States [4].

Living in a food-insufficient household may increase the risk for disordered eating behaviors, including binge eating [5–7]. Food-insufficient individuals may experience periods of “feast or famine” or a time when food intake may decrease due to periods of scarcity and increase due to periods of food abundance (e.g., receiving pay from work) [5]. Restricting food influences a range of psychological and behavioral changes, including increased preoccupation with food and a tendency to binge eat once restrictions are removed [5]. Recurrent binge eating may increase the risk for the development of binge-eating disorder (BED), which is associated with numerous comorbid conditions, including type 2 diabetes and metabolic syndrome [8–10]. In the first study to examine eating disorder pathology among those experiencing food insecurity, those with the highest levels of food insecurity (e.g., adults who reported having hungry children in the household), also endorsed significantly higher levels of binge eating and overall eating disorder pathology [6]. In another study, adults with food insecurity had nearly twice the risk for binge eating than adults who were food-secure, ate breakfast less frequently, and had increased snacking behaviors, consuming 4–5 snacks per day, compared to only 3 to 4 in food-secure households [11].

Adults with food insecurity may also be at increased risk for chronic diseases, including obesity [12, 13]. Indeed, food insecurity is more prevalent among adult women with obesity (impacting 14% vs. 11% of those without the chronic condition), and may increase the risk nearly tenfold for childhood obesity, with stronger associations found in households where mothers also had overweight/obesity [14–17]. Moreover, the risk of obesity may be even greater among adults with food insecurity participating in the Supplemental Nutrition Assistance Program (SNAP)—the largest federal food assistance program in the U.S [17, 18]. In fact, SNAP participants had double the odds for obesity compared to non-participants [19].

Much of what we have learned about food insecurity, binge eating, and obesity have been in samples that are predominately White. Due to disparate rates of food insecurity and obesity in Black households [1, 20], and disparities in treatment access for disordered eating [21], examining the associations among food insecurity, obesity, and binge eating in this population is critical. The Black population of the United States includes approximately 4.5 million immigrants from the Caribbean [22]. Though Afro-Caribbeans share the legacy of slavery with African Americans, they may differ in many other aspects of ethnicity and health outcomes. Thus, we analyzed these groups differently to further evaluate the influence of ethnicity on binge eating and obesity outcomes [23].

## Methods

### Study design and sample

We conducted a secondary analysis of data from the National Survey of American Life (NSAL) [24]. The NSAL is a nationally representative dataset of African Americans (*n* = 3750) and Afro-Caribbeans (*n* = 1623) in the U.S. The NSAL, which was developed as part of the Collaborative Psychiatric Epidemiology Surveys research program, gathered data on the psychological, emotional, and structural and economic conditions facing Black Americans at the beginning of the twenty-first century. The NSAL data were collected between 2001 and 2003 using face-to-face and telephone interviews. The NSAL samples, which were representative of their respective populations, reflected national racial and ethnic distributions on key variables such as education, income, gender, urbanicity, and marital status [24]. Additional information about the design of the NSAL, sample characteristics, sample selection procedures, recruitment and training of interviewers, and methodological limitations are described in Jackson et al. [25]. Missing data reduced our analytical sample size to 4553 (85%). Due to the 1% missing data on non-outcome variables, we did not impute missing data.

### Measures

Sociodemographic variables that were collected include race/ethnicity, age, sex, marital status work status, and years of education.

#### Food sufficiency in the household in the past 12 months

In the NSAL, the presence of food sufficiency in the household was measured through a multiple-choice question asking participants if in the past 12 months, in your household, there was: enough food to eat; sometimes not enough food to eat; or, often not enough food to eat. Single-item measures, similar to the one used in the NSAL, have been validated as useful proxy measures for food sufficiency [26, 27]. We categorized participants as food-insufficient if they often (*n* = 98, 2%) or sometimes (*n* = 399, 9%) did not have enough food to eat, resulting in 497 participants in this category, and food-sufficient if they reported having enough food to eat (*n* = 4056, 89%), as has been done in prior research studies measuring food insufficiency [27, 28].

#### Binge eating

The World Mental Health Composite International Diagnostic Interview (WMH-CIDI) was used to measure binge eating [29]. Trained interviewers assessed binge eating based on DSM-IV criteria for binge-eating episodes in BED. Participants were first asked whether they had experienced eating binges, defined as eating a large amount of food during a short period of time, at least twice a week for several months or longer. Other questions assessed feelings and behaviors during the binge episode, and another question assessed whether eating binges had taken place within the past 12 months. Binge eating over the past 12 months was coded as present if a participant endorsed the first question (i.e., binge eating at least two to three times a week), and one or more of the following to capture the presence of loss of control: (a) eating much more quickly than usual; (b) eating until uncomfortably full; (c) when not physically hungry; (d) eating alone because of feeling embarrassed by the quantity; (e) feeling guilty, depressed, or upset after the binge; (f) often upset that eating was out of control during or after binge; and reported binge eating within the past 12 months. The operationalization of binge eating was based on what has been done in prior research among those using the WMH-CIDI interview [8, 30]. Because of the frequency and duration thresholds used in the survey, we refer to this measure as “recurrent binge eating” to acknowledge it as stricter than simply measuring a binge episode.

#### Obesity

Participants self-reported their height and weight. Based on BMI, participants were classified as having obesity (BMI ≥ 30 kg/m^2^) or not having obesity (BMI < 30 kg/m^2^).

#### Social assistance

NSAL participants were asked if they had received economic assistance from any of the following sources in the past year: social security, worker's compensation, unemployment compensation, food stamps, supplemental security income, earned income tax credit, and child support payments. We recoded the responses to receiving social assistance into three categories: (1) none; (2) one; (3) two or more.

#### Meets all expenses

NSAL participants were asked if, in the past 12 months, there was a time when they (a) did not meet basic needs; (b) did not pay full rent or mortgage; (c) were evicted for non-payment; (d) did not pay full gas or electric, oil; (e) had gas, electric, oil disconnected; (f) had telephone disconnected; (g) could not afford daycare or babysitting; and/or (h) could not afford leisure activities. Responses identifying basic needs not met were recoded into three categories: (1) met all expenses; (2) unable to meet one expense; (3) unable to meet two or more expenses.

#### Dependents

Participants reported the number of children and adolescents in the household, and the sum was used to represent the number of dependents.

#### Household members contributing financially to the household

Participants were asked how many household members, including themselves, gave money to support the household.

### Analysis

We conducted analyses in the overall sample and also stratified by ethnicity and sex. Our choice to stratify was informed by our aim to explore food insufficiency in Black American populations and by known sex differences in the outcomes [31, 32]. We calculated the prevalence of food insufficiency, obesity, and recurrent binge eating and 95% confidence intervals from a binomial distribution. Differences in prevalence among ethnicity and sex subgroups were compared with *z* tests. Chi square analyses and *t* tests were used to assess the association between food insufficiency and sociodemographic, obesity and recurrent binge eating, and economic and poverty indicators. Effect sizes were interpreted according to Cohen’s conventions [33] and were calculated using Cohen’s *d* for continuous variables (small: ≥ 0.2, medium: ≥ 0.5, large: ≥ 0.8) and Cramer’s *V* for categorical variables (*df* = 1: small: ≥ 0.1, medium: ≥ 0.3, large: ≥ 0.5; *df* = 2: small: ≥ 0.07, medium: ≥ 0.21, large: ≥ 0.35; *df* = 3: small: ≥ 0.06, medium: ≥ 0.17, large: ≥ 0.29). We used logistic regression to explore the associations between food insufficiency and obesity and binge eating outcomes. Firth’s bias-reduced logistic regression model was used to predict rare outcomes (i.e., binge eating). We ran unadjusted analyses and analyses adjusted for age, marital status, employment status, and education. The false discovery rate procedure was used to correct for multiple testing [34]. All analyses were conducted using SAS 9.4.

## Results

### Sample characteristics

Participants were 4553 Black Americans of whom 3233 were African American and 1320 were Afro-Caribbean (Table 1). The mean age of the sample was 42 years (*SD* = 16) and approximately two-thirds were female. Marital status was approximately evenly split between married/cohabiting, divorced/separated/widowed, and never married. Most participants were employed (68%) and had at least 12 years of education (76%). Half received social assistance (53%), one third were unable to meet at least one expense (39%), half reported at least one dependent household member (44%) and most participants reported that one (53%) or two (39%) household members supported the household financially.

**Table 1:** Characteristics of National Survey of American Life (NSAL) participants and comparisons by food insufficiency status

Characteristic	Overall sample (*N* = 4553)	Food insufficiency status				
		Food insufficient (*N* = 497)	Food sufficient (*N* = 4056)	*P*	*P*_FDR_	Post hoc
*Sociodemographics*						
Race				0.21	0.23	n/a
African-American	71% (3233)	69% (341)	71% (2892)			
Afro-Caribbean	29% (1320)	31% (156)	29% (1164)			
Age (years): mean ± *SD*	42.37 (16.01)	40.17 (14.25)	42.64 (16.19)	< 0.001***	< 0.001***	n/a
Gender				< 0.001***	< 0.001***	n/a
Male	37% (1705)	29% (146)	38% (1559)			
Female	63% (2848)	71% (351)	62% (2497)			
Marital status				0.001**	0.002**	Divorced/separated/widowed, never married > Married/cohabiting
Married/cohabitating	37% (1705)	29% (143)	39% (1562)			
Divorced/separated/widowed	30% (1346)	33% (163)	29% (1183)			
Never married	33% (1502)	38% (191)	32% (1311)			
Employment status				< 0.001***	< 0.001***	Unemployed > Not in labor force > Employed
Employed	68% (3115)	58% (286)	70% (2829)			
Unemployed	10% (459)	20% (99)	9% (360)			
Not in labor force	22% (979)	23% (112)	21% (867)			
Education				< 0.001***	< 0.001***	0–11 years > 12 years > 13–15 years > 16 + years
0–11 years	24% (1074)	35% (176)	22% (898)			
12 years	35% (1612)	37% (185)	35% (1427)			
13–15 years	24% (1094)	19% (92)	25% (1002)			
16 + years	17% (773)	9% (44)	18% (729)			
*Past 12 months economic/poverty indicators*						
Social assistance				< 0.001***	< 0.001***	Received two or more types > Received one type > Did not receive any
Did not receive any	47% (2160)	33% (162)	49% (1998)			
Received one type	31% (1426)	36% (178)	31% (1248)			
Received two or more types	21% (967)	32% (157)	20% (810)			
Met expenses				< 0.001***	< 0.001***	Did not meet two or more > Did not meet one > Met all
Met all	61% (2788)	24% (120)	66% (2668)			
Did not meet one	15% (670)	14% (71)	15% (599)			
Did not meet two or more	24% (1095)	62% (306)	19% (789)			
Number of household dependents				< 0.001***	< 0.001***	Three or more > None, One, Two
None	56% (2562)	50% (249)	57% (2313)			
One	21% (941)	21% (106)	21% (835)			
Two	13% (587)	13% (64)	13% (523)			
Three or more	10% (463)	16% (78)	9% (385)			
Number of household members who support the household financially				< 0.001***	< 0.001***	
One	53% (2433)	64% (318)	52% (2115)			One > Two, Three or more
Two	39% (1757)	30% (151)	40% (1606)			
Three or more	8% (363)	6% (28)	8% (335)			
*Obesity and binge eating outcomes*						
Obese	33% (1506)	37% (183)	33% (1323)	0.06	0.07	n/a
Recurrent binge eating in the past 12 months	2% (94)	3% (17)	2% (77)	0.02*	0.03*	n/a
Lifetime binge eating	3% (156)	6% (28)	3% (128)	0.004**	0.006**	n/a

The *P*_FDR_ values are corrected for 40 tests in total, comprising omnibus and post hoc tests. The post hoc results column uses a FDR *P* < 0.05 for interpretation

*FDR * False discovery rate

*P < 0.05; **P < 0.01; ***P < 0.001

### Food insufficiency

The prevalence of food insufficiency was 11% among Black Americans (95% CI 10–12%, *n* = 497 of 4553). By ethnicity, the prevalence was 11% among African Americans (95% CI 9–12%, *n* = 341 of 3233) and 12% among Afro-Caribbeans (95% CI 10–14%, *n* = 156 of 1320), and there was no significant difference between these ethnicity groups (χ^2^(1) = 1.56, *P* = 0.21, *P*_FDR_ = 0.24, Cramér’s *V* = 0.02, negligible effect size) (Table 2).

**Table 2:** Prevalence and 95% confidence intervals for food insufficiency, obesity, and recurrent binge eating in Black Americans

Outcome	Black Americans (*N* = 4553)	African American			Afro-Caribbean			Group comparisons									
		Male (*N* = 1170)	Female (*N* = 2063)	Overall (*N* = 3233)	Male (*N* = 535)	Female (*N* = 785)	Overall (*N* = 1320)	Overall African American v overall Afro-Caribbean		Male African American v female African American		Male Afro-Caribbean v female Afro-Caribbean		Male African American v male Afro-Caribbean		Female African American v female Afro-Caribbean	
								*P*	*P*_FDR_	*P*	*P*_FDR_	*P*	*P*_FDR_	*P*	*P*_FDR_	*P*	*P*_FDR_
Food insufficiency	11% (10%, 11%)	8% (7%, 10%)	12% (10%, 13%)	11% (9%, 12%)	9% (6%, 11%)	14% (11%, 16%)	12% (10%, 14%)	0.21	0.29	0.004**	0.01*	0.005**	0.01*	0.82	0.88	0.12	0.18
Obesity	33% (32%, 34%)	28% (26%, 31%)	41% (39%, 43%)	37% (35%, 38%)	17% (14%, 21%)	29% (26%, 33%)	25% (22%, 27%)	< 0.001***	< 0.001***	< 0.001***	< 0.001***	< 0.001***	< 0.001***	< 0.001***	< 0.001***	< 0.001***	< 0.001***
Recurrent binge eating	2% (2%, 2%)	2% (1%, 3%)	3% (1%, 3%)	2% (2%, 3%)	1% (0%, 2%)	2% (1%, 2%)	2% (1%, 2%)	0.10	0.18	0.24	0.30	0.96	0.96	0.57	0.66	0.11	0.18

The *P*_FDR_ values are corrected for 15 tests

*FDR * False discovery rate

*P < 0.05; **P < 0.01; ***P < 0.001

Table 1 presents the sample characteristics by food insufficiency status. Participants experiencing food insufficiency were significantly younger (40 years v 42 years; *t* = 3.60, Satterthwaite *df* = 663.39, *P* < 0.001, *P*_FDR_ < 0.001, Cohen’s *d* = 0.15, negligible effect size) and they were more likely to be female (71% v 62%; χ^2^(1) = 15.52, *P* < 0.001, *P*_FDR_ < 0.001, Cramér’s *V* = 0.06, negligible effect size). They were also more likely to be divorced (33% v 29%; χ^2^(1) = 11.55, *P* < 0.001, *P*_FDR_ = 0.001, Cramér’s *V* = 0.05, negligible effect size) or never married (38% v 32%; χ^2^(1) = 16.04, *P* < 0.001, *P*_FDR_ = 0.001, Cramér’s *V* = 0.06, negligible effect size) and to be unemployed (20% v 9%; χ^2^(1) = 63.86, *P* < 0.001, *P*_FDR_ < 0.001, Cramér’s *V* = 0.12, small effect size) or not in the labor force (23% v 21%; χ^2^(1) = 4.33, *P* = 0.04, *P*_FDR_ = 0.04, Cramér’s *V* = 0.03, negligible effect size). As years of education decreased the likelihood of food insufficiency increased, for instance, post hoc tests showed that those with the lowest level of education were significantly more likely to be food insufficient (16%) than those with 12 years (11%), 13–15 years (8%), and 16 + years (6%) of education (see Table 1). Participants experiencing food insufficiency were also more likely to have received one (36% v 31%; χ^2^(1) = 24.85, *P* < 0.001, *P*_FDR_ < 0.001, Cramér’s *V* = 0.07, negligible effect size) or two types of social assistance (32% v 20%; χ^2^(1) = 55.64, *P* < 0.001, *P*_FDR_ = 0.001, Cramér’s *V* = 0.11, small effect size), and those on two types had a higher rate of food insufficiency than those on one type (16% v 12%; χ^2^(1) = 6.74, *P* = 0.009, *P*_FDR_ = 0.01, Cramér’s *V* = 0.04, negligible effect size). Food insufficient participants were also more likely to be unable to meet one (14% v 15%; χ^2^(1) = 40.99, *P* < 0.001, *P*_FDR_ < 0.001, Cramér’s *V* = 0.09, negligible effect size) or two expenses (62% v 19%; χ^2^(1) = 449.88, *P* < 0.001, *P*_FDR_ < 0.001, Cramér’s *V* = 0.31, medium effect size) compared to having met all expenses (24% v 66%), and those who were unable to meet two expenses had higher levels of food insufficiency than those who were unable to meet one expense (χ^2^(1) = 74.48, *P* < 0.001, *P*_FDR_ < 0.001, Cramér’s *V* = 0.13, small effect size). Participants with many dependents (i.e., three or more) were more likely to be food insufficient (16%, χ^2^(1) = 20.66, *P* < 0.001, *P*_FDR_ < 0.001, Cramér’s *V* = 0.07, negligible effect size) compared to those with no dependents (50%), and participants reporting only one household member supporting the household were more likely to be food insufficient (64%) than participants reporting two (30%; χ^2^(1) = 20.56, *P* < 0.001, *P*_FDR_ < 0.001, Cramér’s *V* = 0.07, negligible effect size), or three or more members (6%; χ^2^(1) = 8.36, *P* = 0.003, *P*_FDR_ = 0.01, Cramér’s *V* = 0.04, negligible effect size).

The prevalence of obesity did not differ significantly between participants who were food insufficient and food sufficient (37% v 33%; χ^2^(1) = 3.53, *P* = 0.06, *P*_FDR_ = 0.07, Cramér’s *V* = 0.03, negligible effect size). Individuals with food insufficiency were more likely than individuals with food sufficiency to report recurrent binge eating in the past 12 months (3% v 2%; χ^2^(1) = 5.07, *P* = 0.02, *P*_FDR_ = 0.03, Cramér’s *V* = 0.03, negligible effect size) and a lifetime history of recurrent binge eating (6% v 3%; χ^2^(1) = 8.22, *P* = 0.004, *P*_FDR_ = 0.006, Cramér’s *V* = 0.04, negligible effect size).

Sex-specific prevalence estimates are shown in Table 2. Food insufficiency prevalence was 12% (95% CI 10–13%, *n* = 242 of 2063) in African American females and 8% (95% CI 7–10%, *n* = 99 of 1170) in African-American males, and 14% (95% CI 11–16%, *n* = 109 of 785) in Afro-Caribbean females and 9% (95% CI 6–11%, *n* = 47 of 535) in Afro-Caribbean males. These sex differences in prevalence estimates within both racial/ethnic groups were statistically significant (*P*_FDR_*s* < 0.05).

### Prevalence of obesity and binge eating

The prevalence of obesity was 33% (95% CI 31–34%, *n* = 1506 of 4553) among Black Americans, and specifically, 37% (95% CI 35–38%, *n* = 1182 of 3233) among African Americans and 25% (95% CI 22–27%, *n* = 324 of 1320) among Afro-Caribbeans. Obesity was 1.5 times more common among African-Americans than Afro-Caribbeans (*z* = − 7.82, *P* < 0.001, *P*_FDR_ < 0.001), and was significantly more common among females than males in African-Americans (*z* = − 7.28, *P* < 0.001, *P*_FDR_ < 0.001) and Afro-Caribbeans (*z* = − 4.99, *P* < 0.001, *P*_FDR_ < 0.001) (Table 2). The prevalence of recurrent binge eating was 2% (95% CI 1–2%, *n* = 94 of 4553) among Black Americans. By subgroup, the prevalence was 2.3% (95% CI 1.8–2.8%, *n* = 74 of 3233) among African Americans and 1.5% (95% CI 0.9–2.2%, *n* = 20 of 1320) among Afro-Caribbeans (Table 2). Recurrent binge eating prevalence did not differ significantly between African Americans and Afro-Caribbeans (*z* = − 1.67, *P* = 0.10, *P*_FDR_ = 0.18) and there were no statistically significant sex differences (Table 2).

### Ethnicity, food insufficiency, and obesity

Table 3 presents the associations among ethnicity, food insufficiency, and obesity. There were no significant associations between food insufficiency and obesity in the unadjusted models. After adjusting for age, marital status, education, and employment status, food insufficiency increased the odds of obesity in Afro-Caribbeans before but not after correction for multiple testing (odds ratio [OR] = 1.47, 95% CI 1.01, 2.13, *P* = 0.04, *P*_FDR_ = 0.25). Food insufficiency was not significantly associated with obesity in African Americans (1.16, 95% CI 0.92, 1.47, *P* = 0.22, *P*_FDR_ = 0.33) or in the overall Black American sample (1.20, 95% CI 0.99, 1.46, *P* = 0.07, *P*_FDR_ = 0.25).

**Table 3:** Food insufficiency as a predictor of obesity among Black Americans

Model	Black Americans (*N* = 4553)			African American									Afro-Caribbean								
				Male (*N* = 1170)			Female (*N* = 2063)			Overall (*N* = 3233)			Male (*N* = 535)			Female (*N* = 785)			Overall (*N* = 1320)		
	OR (95% CI)	*P*	*P*_FDR_	OR (95% CI)	*P*	*P*_FDR_	OR (95% CI)	*P*	*P*_FDR_	OR (95% CI)	*P*	*P*_FDR_	OR (95% CI)	*P*	*P*_FDR_	OR (95% CI)	*P*	*P*_FDR_	OR (95% CI)	*P*	*P*_FDR_
Unadjusted	1.20 (0.99, 1.46)	0.06	0.25	0.84 (0.52, 1.35)	0.47	0.51	1.22 (0.93, 1.59)	0.15	0.33	1.16 (0.92, 1.45)	0.22	0.33	1.51 (0.74, 3.09)	0.26	0.33	1.28 (0.83, 1.96)	0.27	0.33	1.43 (0.99, 2.06)	0.06	0.25
Adjusted	1.20 (0.99, 1.46)	0.07	0.25	0.90 (0.56, 1.45)	0.67	0.67	1.21 (0.92, 1.59)	0.17	0.33	1.16 (0.92, 1.47)	0.20	0.33	1.50 (0.72, 3.14)	0.28	0.33	1.32 (0.85, 2.05)	0.21	0.33	1.47 (1.01, 2.13)	0.04*	0.25

The referent for food insufficiency is food sufficiency. The adjusted model adjusts for age, marital status, education, and employment status.

The *P*_FDR_ values are corrected for 14 tests

*FDR * False discovery rate

*P < 0.05.

### Ethnicity, food insufficiency, and binge eating

Table 4 reports the associations among ethnicity, food insufficiency, and recurrent binge eating. In the unadjusted model, food insufficiency significantly increased the odds of recurrent binge eating in the overall sample of Black Americans before but not after adjusting for multiple testing (1.87, 95% CI 1.10, 3.17, *P* = 0.02, *P*_FDR_ = 0.16). In addition, when examining this relationship by gender and ethnicity, food insufficiency significantly increased the odds of recurrent binge eating in Afro-Caribbean women (3.35, 95% CI 1.05, 10.75, *P* = 0.04, *P*_FDR_ = 0.16) and in the overall Afro-Caribbean sample (2.69, 95% CI 1.00, 7.24, *P* = 0.04, *P*_FDR_ = 0.16) before but not after correction for multiple testing, and did not increase the odds of recurrent binge eating in African Americans (1.72, 95% CI 0.92, 3.19, *P* = 0.09, *P*_FDR_ = 0.16). After adjusting for age, marital status, education, and employment status, the odds ratios attenuated and food insufficiency no longer significantly increased the odds for recurrent binge eating in either ethnicity group. The model output suggested that lower age was associated with higher binge eating (*P*_FDR_s < 0.05).

**Table 4:** Food insufficiency as a predictor of recurrent binge eating among Black Americans

Model	Black Americans (*N* = 4553)			African American									Afro-Caribbean								
				Male (*N* = 1170)			Female (*N* = 2063)			Overall (*N* = 3233)			Male (*N* = 535)			Female (*N* = 785)			Overall (*N* = 1320)		
	OR (95% CI)	*P*	*P*_FDR_	OR (95% CI)	*P*	*P*_FDR_	OR (95% CI)	*P*	*P*_FDR_	OR (95% CI)	*P*	*P*_FDR_	OR (95% CI)	*P*	*P*_FDR_	OR (95% CI)	*P*	*P*_FDR_	OR (95% CI)	*P*	*P*_FDR_
Unadjusted	1.87 (1.10, 3.17)	0.02*	0.16	1.31 (0.35, 5.00)	0.69	0.74	1.89 (0.95, 3.77)	0.07	0.16	1.72 (0.92, 3.19)	0.09	0.16	2.07 (0.34, 12.46)	0.43	0.50	3.35 (1.05, 10.75)	0.04*	0.16	2.69 (1.00, 7.24)	0.04*	0.16
Adjusted	1.58 (0.93, 2.69)	0.09	0.16	1.11 (0.30, 4.10)	0.87	0.87	1.57 (0.79, 3.14)	0.20	0.31	1.45 (0.78, 2.70)	0.24	0.34	2.52 (0.47, 13.58)	0.28	0.36	2.67 (0.84, 8.43)	0.09	0.16	2.60 (0.97, 6.94)	0.06	0.16

The referent for food insufficiency is food sufficiency. The adjusted model adjusts for age, marital status, education, and employment status. The *P*_FDR_ values are corrected for 14 tests

*FDR * False discovery rate

*P < 0.05

## Discussion

This study examined the relation among food insufficiency, obesity, and recurrent binge eating in a nationally-representative sample of African Americans and Afro-Caribbeans. The results of this study provide further evidence to confirm the disparity of obesity among African Americans, specifically that of African American women, that has been reported for nearly two decades [35, 36]. Additionally, in the overall sample, participants who reported experiencing food insufficiency had increased odds of recurrent binge eating in the past 12 months, and a lifetime history of recurrent binge eating, but not obesity. After adjusting for various socio-demographic factors and using FDR, food insufficiency was no longer significantly associated with recurrent binge eating. We did observe that lower age was associated with recurrent binge eating. It is critical that researchers consider the environmental dynamics behind the experience of poverty and risk for binge eating and obesity. Future research should explore factors contributing to increased binge eating and obesity among those living in a food-insufficient household, and uncover additional reasons for the continued disparity in obesity rates. Additionally, future research should investigate whether food insufficiency is associated with a lower threshold of binge eating, since this study used a strict measure of binge eating frequency compatible with a DSM-IV diagnosis of BED (i.e., at least twice per week). Less strict measures would include simply an episode of binge eating, with a minimum frequency of once per week (DSM-5 threshold), and a lower duration.

### Prevalence of food insufficiency

The results of this study suggest that nearly 12% of African Americans and Afro- Caribbeans reported experiencing food insufficiency. Additionally, those with food insufficiency were more likely to be female, with three or more dependents, and with few household members contributing financially to the household. This adds to what has been observed in the growing scientific literature on food insufficiency; when examining racial/ethnic differences in rates of food insufficiency, researchers have observed a higher prevalence in African American/Black populations and in households that are headed by single mothers [37, 38]. Future research should continue to investigate the racial disparities in food insufficiency prevalence, and relevant contributing factors.

### Prevalence of obesity and binge eating

One goal of our study was to determine whether the relations among food insufficiency, obesity, and binge eating differed between African American and Afro-Caribbean participants. In this study, Afro-Caribbean participants had significantly lower obesity prevalence than African Americans (25% vs. 37%). The most recent estimates indicate that obesity prevalence estimates among Non-Hispanic Black adults, at nearly 50%, are the highest in the United States [20]. Moreover, when examining prevalence of obesity by sub-group, African American women have significantly higher prevalence of obesity (57%), compared to African American men (41%), Hispanic women (44%), and Non-Hispanic White women (40%) [20].

In contrast, in this study, African American and Afro-Caribbean participants reported approximately equal prevalence of recurrent binge eating (approximately 2%). Previous studies have reported a wide range of binge eating prevalence in Black Americans, with estimates ranging from 1.5 to 36% [39]. It is noteworthy these binge eating behaviors were self-reported vs. captured with an interview-based measure often used to assess episodes of binge eating (e.g., Eating Disorder Examination) [40]. The strict measure of binge eating used in this study may have caused the captured prevalence rates to be lower. Although self-reported binge eating is typically higher than interviewer-assessed binge eating in predominantly White samples, the level of self-reported binge eating in this study may also reflect the lack of awareness of binge eating that has been observed among Black Americans [41], further impacting documented treatment disparities. Indeed, African American/Black adults are far less likely to access traditional forms of treatment for disordered eating [39, 42].

### Food insufficiency and obesity

In this study, the prevalence of obesity did not differ between food-sufficient and food-insufficient participants. Similarly, Vedovato et al. [43] did not find an association between food insecurity and obesity in a sample of 298 low-income, African American families. This, however, is contrary to what has been observed in previous studies in other racial and ethnic groups. For example, food insecurity has been associated with a 41% higher odds of obesity in White women, and a 29% higher odds of obesity in Latino women [37]. Similarly, food insecurity is significantly associated with obesity in Mexican–American women, but not Mexican–American men [44]. Our findings may reflect the prevalence of overweight and obesity in the entire sample, such that food insufficiency status may not be as impactful. Alternatively, there may be a chance some participants only experienced mild to moderate levels of food insufficiency; this may have impacted our ability to accurately measure the association with obesity. Future research should continue to examine these associations in additional samples of Black Americans to examine if these results may vary with levels of food insufficiency (e.g., very low, low), the addition of other environmental factors (e.g., residing in a food desert and/or food swamp), or other socio-economic considerations.

The results of this study also confirm that receiving multiple forms of social assistance (e.g., social security, worker's compensation, unemployment compensation, SNAP or food stamp benefits) is associated with increased risk of food insufficiency. Receiving multiple forms of social assistance may serve as a proxy for poverty, and may also reflect the experiences of households with extreme levels of food insufficiency. Growing research has highlighted how participating in the Supplemental Nutrition Assistance Program (SNAP) is positively associated with obesity, particularly among women [45–50]. The “food-stamp cycle”, a 3-week period where SNAP benefits and money are available, and followed by a 1-week period where SNAP benefits and resources are limited might contribute to this risk; [51] participants may restrict their diets during the period when food is limited, and then overeat once SNAP benefits are restored**;** these chronic patterns of undereating and overeating may impact weight gain [11, 51, 52]. Moreover, researchers have suggested the need for program changes to encourage the consumption of high-nutrient and lower-calorie foods [50]. Further research should identify additional factors that affect eating choices and patterns among those who receive multiple forms of social assistance to inform future policy interventions.

### Black ethnicity, food insufficiency, and binge eating

In this study, individuals with food-insufficiency were more likely to report recurrent binge eating and lifetime binge eating; however, this relationship became non-significant after correcting for multiple testing. To explore this further, it would be important in future research to examine this association by level of food insufficiency. A growing research literature has drawn attention to how experiencing recurrent food shortages increases the likelihood for periods of under and over-eating, and maladaptive eating experiences [5, 6, 47, 52]. This risk may vary depending on level of food insufficiency experienced; in fact, among samples of participants experiencing the most extreme levels of food insufficiency, the risk for BED and obesity was elevated [5, 6].

### Black ethnicity, obesity, and binge eating

Within this study, ethnicity moderated the relationship between food insufficiency and obesity and, food insufficiency and recurrent binge eating in uncorrected analyses, though the relationship was no longer significant after adjusting for multiple testing and sociodemographic factors. Notable differences include the fact that among Afro-Caribbeans only, individuals who were experiencing food insufficiency had increased odds for binge eating and obesity. In a 2017 study, researchers observed similar results when examining cross-sectional data from the 2011–2012 National Health Interview Survey; food insecurity was not related to overweight/obesity in African Americans [37]. Food insecurity also has not been associated with type 2 diabetes, a known and common obesity-related co-occurring condition in African Americans [53]. We also observed significantly higher rates of obesity in African Americans than Afro-Caribbeans. Although the reasons for this ethnic difference are likely varied and complex, we can infer that these differences do represent the variations that may be present across cultural and psychosocial profiles found in Black ethnic groups. For example, scholars have noted that less acculturation and high levels of familism among Afro-Caribbeans may explain lower obesity prevalence [54]. Further, previous research has documented that Afro-Caribbean adults were more likely than African Americans to live in neighborhoods with a supermarket or a park, measures both previously associated with obesity in these sub-groups [55]. Overall, the results of this study may further highlight the diversity that is present within Black Americans and warrants additional study to prevent and reduce health disparities, and improve general understanding of the experiences of this heterogeneous and expanding population in the United States. Further, our results underscore the importance of making distinctions between African American and Afro-Caribbean participants in research as combining them under the umbrella of “Black Americans” could obscure important group differences.

### Implications for practice and policy

The results of this study contribute further evidence to aid in expanding our understanding of the cultural intersections for Black Americans, food insecurity, obesity and binge eating. Clinicians have acknowledged challenges assessing and recognizing disordered eating behavior in this population [56]. In addition to assessing for food insufficiency in the treatment of eating disorders and obesity, clinicians would also benefit from conducting culturally-relevant assessments, making sure to incorporate factors known to influence the eating behaviors of Black Americans, including but not limited to perceived discrimination, trauma, and poverty [39, 57, 58]. A growing evidence base highlights how the experience of poverty may have certain risk factors (e.g., periods of feast or famine) that increase disordered eating [6, 7]; Understanding if patients have all of their basic needs met, and who may or may not be experiencing periods of food shortage is critical.

Additionally, the results of this paper add to a growing database highlighting the need to reevaluate policy decisions to care for those experiencing food insufficiency, due to evidence for the risk for disordered eating. Though the restriction is externally motivated, rather than the internal desire to achieve thinness, the binge eating experienced by those living in a food-insufficient household may indeed be a response to cyclical periods of food shortage [5]. Scholars have noted the challenges of federal programs for those experiencing food insufficiency, noting that systems could be adjusted to better accommodate the realities of living with food insufficiency [5, 47, 50]. For example, the SNAP program, currently administers benefits once per month. With increased rates of binge eating and obesity among participants in this program, future policy change could examine the benefit of staggered allocation of food benefits to potentially alleviate the strain of cyclical food deprivation [5]. Further among a qualitative study of food insufficient participants, recommendations were made to increase use of policies that facilitate purchase of healthy foods, and limit purchase of nonhealthy foods, with a particular emphasis on sugary beverages [59].

This study has several limitations. The data used to interpret the relation among recurrent binge eating, obesity, and food insufficiency were collected cross-sectionally. Data collection was nearly 20 years ago, and may not fully encompass the extent of the most recent relationship among these variables. Data on food insufficiency was limited in the NSAL sample, and the extent and/or level of food insufficiency was not reported. Additionally, due to small cell sizes, we were unable to examine the relationship between the variables for those who reported the very highest levels of food insufficiency (approximately 2% of sample). Moreover, because of the limitations of using existing data, we were unable to explore the relevance of food insecurity, in addition to food insufficiency, because it was not measured. Participants also self-reported height and weight, thereby increasing the likelihood of inaccurate reporting, and limiting interpretation of obesity classification. Additionally, our measure of binge eating and our results may be directly relevant to a DSM-IV diagnosis for binge-eating disorder. Because the DSM-5 decreases the required days of binge eating to 1 day per week/3 months, our results may be more representative of describing the relationship between food insufficiency and an individual who is suffering from moderate to severe binge eating. If the threshold for binge eating behavior was lower, we may have seen a larger percentage of food-insufficient individuals with the disordered eating behavior. Further, although it is likely that many of the participants identified with recurrent binge eating in this study likely experienced loss of control during their binge episodes, this cannot be verified; Loss of control during binge eating was not explicitly assessed in this study. Nevertheless, this study is one of the first to investigate the associations among food insufficiency, recurrent binge eating, and obesity in a nationally-representative sample of Black participants. Furthermore, this study provides evidence to further scientific knowledge about these factors in a marginalized population, thereby extending our knowledge, and providing further evidence to assist researchers and policy makers in developing appropriate interventions.

## Conclusion

The results of this study contribute to further scientific knowledge about the relation among food insufficiency, obesity, and recurrent binge eating in a nationally-representative sample of African American and Afro-Caribbean adults. Future research should examine the longitudinal nature of this relationship, making sure to include a racially/ethnically diverse sample, distinguishing between African American and Afro-Caribbean participants, measuring levels of food insufficiency, and using interviewer-verified measures of binge eating and obesity that conform with the latest nomenclature.

## Data Availability

The NSAL dataset was used in this manuscript. This data is publicly available, and can be downloaded from Interuniversity Consortium for Political and Social Research (ICPSR), Institute for Social Research at the University of Michigan: https://www.icpsr.umich.edu/web/RCMD/studies/36380.
